# Medical Treatment of Children and Youths with Attention-Deficit/Hyperactivity Disorder (ADHD): A Norwegian Prescription Registry Based Study

**DOI:** 10.5539/gjhs.v6n4p155

**Published:** 2014-04-14

**Authors:** Jan Norum, Aina Iren Olsen, Frank Ivar Nohr, Anca Heyd, Arpad Totth

**Affiliations:** 1 Institute of Clinical Medicine, Faculty of Health Sciences, University of Tromsø - The Arctic University of Norway, Tromsø, Norway; 2 Department of Radiology, University Hospital of North Norway, Tromsø, Norway; 3 Northern Norway Regional Health Authority Trust, Bodø, Norway; 4 Csolnoky Ferenc Hospital, Veszprém, H-8200 Veszprém, Hungary

**Keywords:** ADHD, Norway, prescription

## Abstract

**Objectives:**

Attention-deficit/hyperactivity disorder (ADHD) is a lifelong neurological condition with a profound effect on quality of life. Prescription databases may document pattern of use. In this study we aimed to explore the use in Norway employing such a database.

**Methods:**

All prescriptions on drugs for the treatment of ADHD between 2004 and 2011, as registered in the Norwegian Prescription Database (NPD) were analyzed. The following drugs were included: Amphetamine, dexamphetamine, methylphenidate and atomoxetine. In-hospital drug administration was excluded. Numbers of users per 1,000 inhabitants were calculated according to gender, age and residence. A sub-analysis compared users born in January-June with those born in July-December. Drug costs were calculated and converted into Euros (€ 1 = N.kr 7.4540).

**Results:**

Drugs for the treatment of ADHD was significantly more often prescribed in northern Norway than in any other Norwegian health region (P < 0.001). Within the northern region, Nordland County was the “culprit” (P < 0.02). Compared to Norwegian figures, significantly more females (aged 10-19 years) were treated in northern Norway [male/female ratios 3:1 and 2.2:1 (P < 0.01)] and especially in Nordland County (ratio 2.1:1). The subanalysis did not indicate a northern overtreatment of those being a younger group in their grade. The annual drug cost per user in Norway was € 919.

**Conclusions:**

The prescription rate was significantly higher in northern Norway and Nordland County was the culprit. A prescription database may be a tool for monitoring the national use of these drugs.

## 1. Introduction

Attention-deficit/hyperactivity disorder (ADHD) is a neuropsychiatric condition characterized by core symptoms of inattentiveness, hyperactivity, and impulsivity (Harpin, 2005). It is potentially a lifelong condition with a profound effect on quality of life (Shaw et al., 2012; National Institute of Health and Clinical Excellence, 2009; American Academy of Pediatrics, 2000; Coghill et al., 2009; Ebert et al., 2003; Pliszka, 2007). Research based on the International Classification of Diseases revision 10 (ICD-10) has suggested an incidence rate of 1-3% of the population. Among school aged children figures have been reported between 3-5% (Biederman et al., 2004). Internationally, the male/female ratio has been reported 4:1 (MTA, 1999). The rise of ADHD diagnoses and prescriptions for stimulants over the years has coincided with a remarkably successful two-decade campaign by pharmaceutical companies to publicize the syndrome and promote the pills to doctors, educators and parents. The zeal to find and treat every ADHD child may have led to too many people with scant symptoms receiving the diagnosis and medication. The disorder is now the second most frequent long-term diagnosis made in children, narrowly trailing asthma (Schwartz, 2013).

Medical treatment may reduce the negative impact that untreated ADHD has on life functioning, but does not usually normalize the recipients (Shaw et al., 2012). The multimodal treatment study of children with ADHD has documented the effect of various treatments, including intensive behavioral intervention, medication and combinations or routine community care (MTA, 1999). Overall treatment of ADHD resulted in favorable outcomes for most outcomes reported (Shaw et al., 2012). Children should have a similar access to health care services regardless of age, gender or place of living. Consequently, the quality of clinical investigation, diagnostics and treatment of ADHD must be similar throughout Norway. However, data from the Norwegian Patient Registry (NPR) has indicated variations in the numbers of children and youths treated for ADHD between Norwegian health regions. Compared to national average figures, higher numbers has been reported in the northern region (Surén et al., 2013). Fewer patients treated in the primary health care, a higher incidence rate and/or over-diagnosis has been plausible explanations. To clarify the possible influence of level of care (primary or specialized health care) and possible over-treatment, we initiated a prescription database study.

## 2. Material and Methods

Patients with ADHD may be treated in the primary and specialized health care. Consequently, a high frequency of care in the specialized health care (SHC) in one region may simply be due to less activity in the primary health care (PHC) and vice versa. To clarify the total medical treatment of ADHD, we employed the Norwegian Prescription Database (NPD). Medical treatment of ADHD can be monitored employing the NPD (www.norpd.no) as the registry includes all drugs prescribed in Norway to Norwegian patients.

All prescriptions on drugs for the treatment of ADHD were included in the study and the eight years time period between January 1^st^ 2004 and December 31^st^ 2011 was analyzed. The following drugs were included: Amphetamine, dexamphetamine, methylphenidate and atomoxetine. Data on in-hospital administration was not included as no individual prescription was employed in this setting. As patients may employ/test several drugs repeatedly for ADHD during a year, we did not perform any sub-analysis for each drug.

**Figure 1:**
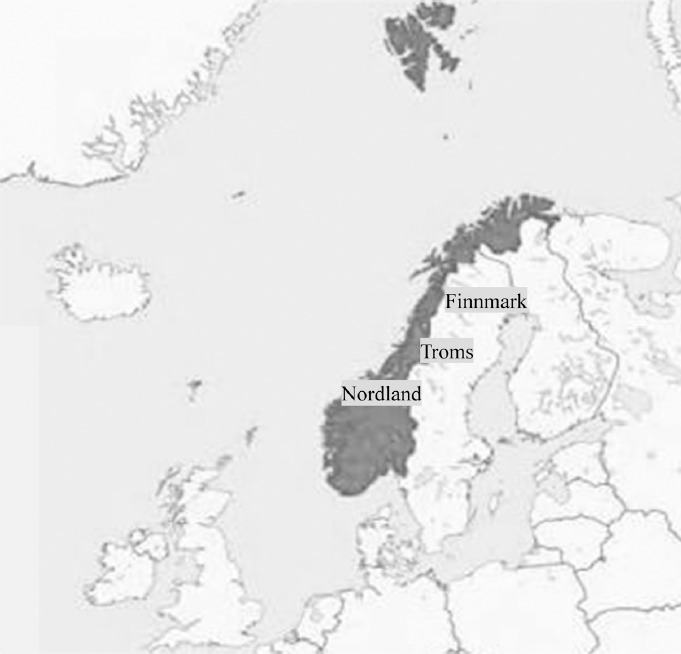
The figure shows Norway (in dark colour) and the three northern counties named Finnmark, Troms and Nordland

An ADHD drug user was defined as a person who had at least one prescription dispensed in a pharmacy each year. The number of users per year was calculated in all four health regions (Southeastern, Western, Central and Northern) and in the three counties of Northern Norway (Finnmark, Troms and Nordland). The location of Norway at the top of Europe and its three northern counties are visualised in Figure 1. The numbers of users was calculated as numbers per 1,000 inhabitants in each age group. Sub-analysis was based on gender, age, month of birth and residence groups. Furthermore, we did also calculate the annual cost of these drugs employing the pharmacy retail price in Norwegian kroner (N.kr) and converted into Euro (€) at the rate of € 1 = N.kr 7.4540 as of the 27^th^ of March 2013 (www.norges-bank.no).

ADHD is an underlying neurological problem and consequently incidence rates should not vary from one birth date to the next. Children born during the second half of the year are a younger group in their grade and the resulting differences in behaviour may lead schoolteachers and health care workers to misinterpret immaturity as symptoms of ADHD. Consequently, a risk of overdiagnosis and overtreatment may occur. To clarify this risk, we sought to indicate the quality of care in terms of the influence of relative age within a grade on the pharmacologic treatment of ADHD. The users were divided into two subgroups and analyzed. Group A consisted of users born between January 1^st^ and June 30^th^ and group B of users born between July 1^st^ and December 31^st^.

### 2.1 Statistical Analysis and Authorisation

In the NPD every individual is allocated a serial number, a so-called pseudonym. This makes it possible to link drug use to individuals and follow their consumption over time, without knowing their identities. Consequently, a person who has collected numerous prescriptions for the same drug is only counted once per year. An inhabitant’s residence was defined as their place of residence on January 1^st^ each year. The number of users was calculated annually and per 1,000 inhabitants of actual age group. Calculations were performed by the NPD and cohorts based on national data on sex, age cohorts (5 years intervals) and place of living were employed.

We accessed anonymous and aggregated data from the NPD. Users without a Norwegian personal identification number were excluded. Consequently any non-Norwegian persons given a prescription of an ADHD pharmacotherapy was excluded from the study. The aggregated data were imported into a database at the NNRHA. Microsoft Excel 2007 version was employed for the final database, calculations and statistical analysis. The comparison between regions/counties with regard to users and costs were based on rates. Descriptive statistics and t-test were used for the comparisons. The t-test was used in comparison of incidence rates and a two-sample equal variance (homoscedastic) test was used. Significance was set to 5%. The t-test was carried out two-sided. Most data from the NPD was available on the Web free of cost, but we did also purchase (€ 805) extracted anonymous data of users according to month of birth (first or last six months of the year). As we imported only aggregated data, no ethical committee or Data Inspectorate approval was necessary. Consequently no approval from the Regional Committees for Medical and Health Research Ethics (REK) was necessary. Similarly, no approval from the Norwegian Social Science Data Services (NSD) was required.

## 3. Results

**Figure 2:**
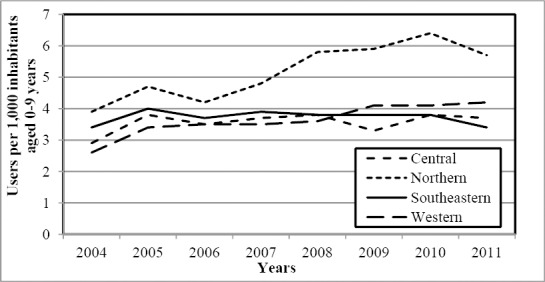
The annual number of users of drugs for ADHD aged 0-9 years per 1,000 inhabitants

**Figure 3:**
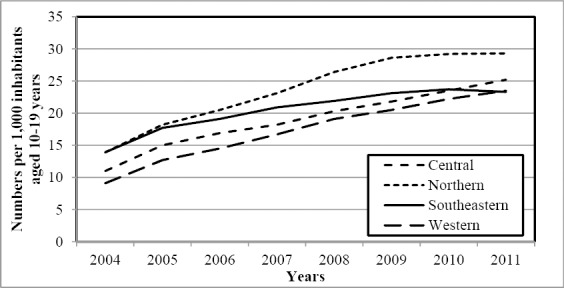
The annual number of users of drugs for ADHD aged 10-19 years per 1,000 inhabitants

Drugs for ADHD were significantly more often prescribed to children and youths in northern Norway (P < 0.001). There were 44% and 25% more users in the northern region than in Norway in the age groups 0-9 years (Figure 2) and 10-19 years (Figure 3), respectively. The significant difference was observed in both genders.

Details are shown in Table 1. In total, the western region had the lowest number of users during study period. The difference reached significant significance in the age group 10-19 years (P < 0.002). However, it should be noted that the gap between the western and the combined southeastern and central regions was closed at the end of the study period. Details are shown in Figure 3.

**Table 1:** The table shows the number of users of drugs for ADHD according to gender in two age cohorts in Norway

Region	Users per 1,000 inhabitants											t-test P-value

	Age	Sex	2004	2005	2006	2007	2008	2009	2010	2011	Mean	
Central	0-9	F	1,1	1,8	1,6	1,6	1,8	1,4	2,0	1,9	1,6	< 0,003*
Northern	0-9	F	1,6	1,9	1,8	2,0	2,8	2,7	2,5	2,2	2,2	-
Southeastern	0-9	F	1,3	1,6	1,5	1,7	1,7	1,8	2,0	1,7	1,7	< 0,001*
Western	0-9	F	0,9	1,3	1,3	1,4	1,7	1,9	1,8	2,0	1,5	< 0,001*
Central	0-9	M	4,7	5,7	5,4	5,7	5,7	5,2	5,6	5,4	5,4	< 0,001*
Northern	0-9	M	6,0	7,3	6,6	7,4	8,6	9,0	10,0	9,0	8,0	-
Southeastern	0-9	M	5,4	6,2	5,8	6,1	5,8	5,7	5,4	5,1	5,7	< 0,002*
Western	0-9	M	4,2	5,5	5,6	5,6	5,4	6,3	6,3	6,4	5,7	< 0,001*
Central	10-19	F	4,3	6,5	7,7	9,2	10,2	11,4	11,9	13,7	9,3	< 0,001*
Northern	10-19	F	6,7	9,7	11,7	14,1	17,1	19,6	19,8	18,7	14,7	-
Southeastern	10-19	F	5,8	8,4	10,1	11,5	12,5	13,3	13,8	13,7	11,1	< 0,001*
Western	10-19	F	3,4	5,5	6,9	8,4	10,1	11,4	13,1	13,9	9,1	< 0,001*
Central	10-19	M	17,4	23,2	25,8	26,9	29,9	31,7	34,4	36,1	28,2	< 0,001*
Northern	10-19	M	20,5	26,0	28,7	31,6	35,1	36,9	37,9	39,1	32,0	-
Southeastern	10-19	M	21,5	26,5	27,8	29,8	30,9	32,4	32,9	32,4	29,3	< 0,015*
Western	10-19	M	14,6	19,4	21,6	24,6	27,7	29,1	30,7	32,5	25,0	< 0,001*

**Figure 4:**
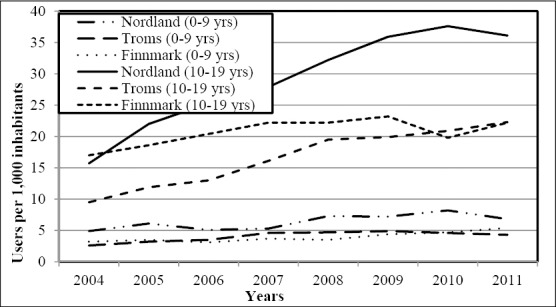
The annual number of users (per 1,000 inhabitants) of drugs for ADHD aged 0-9 years and 10-19 years according to county in northern Norway

To elucidate the higher figures revealed in the northern region, we analyzed figures of the three counties of northern Norway (Nordland, Troms and Finnmark). Nordland County was clearly the “culprit”. The standardized number of users was significantly higher in this county compared with Finnmark County (P < 0.001) and Troms County (P < 0.001). Details are shown in Figure 4.

Looking at male/female ratio, boys dominated the age group 0-9 years by 4:1 and there were no differences between regions. In the age group 10-19 years, the male/female ratio was significantly lower in the northern region (P < 0.01) (2.2:1 versus 3:1). Again Nordland County was the “culprit” [Nordland 2.1/1, Troms 2.3/1 (P < 0.04), Finnmark 2.5/1 (P < 0.02)]. Consequently, the more common use of ADHD drugs in northern Norway within the age group 10-19 years was especially observed among females and in Nordland County. Details are shown in Table 2.

**Table 2:** The table shows the use of drugs for ADHD according to gender in two age cohorts in counties of northern Norway. When five or less users were detected, no information was given and marked with #

County	Users per 1,000 inhabitants											t-test P-value

	Age	Sex	2004	2005	2006	2007	2008	2009	2010	2011	*Mean*	
Nordland	0-9	F	2.1	2.5	2.1	2.4	3.5	3.2	3.4	3.0	*2.8*	-
	0-9	M	7.6	9.6	8.0	8.1	10.9	10.9	12.6	10.4	*9.8*	-
Troms	0-9	F	1.1	1.5	2.0	1.7	2.6	2.4	1.3	0.9	*1.7*	< 0.004*
	0-9	M	4.0	4.8	4.9	7.2	6.7	7.3	7.7	7.4	*6.3*	< 0.001*
Finnmark	0-9	F	#	1.3	#	1.4	#	1.7	2.2	2.5	*1.8*	< 0.003*
	0-9	M	5.3	5.5	5.7	5.9	6.0	7.0	7.2	8.2	*6.3*	< 0.001*
Nordland	10-19	F	8.0	12.3	15.0	16.9	20.7	25.4	26.7	24.7	*18.7*	-
	10-19	M	22.8	31.0	34.9	38.2	43.1	45.7	47.8	46.9	*38.8*	-
Troms	10-19	F	4.8	6.5	7.3	10.0	12.3	12.5	13.0	12.7	*9.9*	< 0.001*
	10-19	M	13.9	16.9	18.4	21.7	26.1	26.5	28.1	31.0	*22.8*	< 0.001*
Finnmark	10-19	F	6.8	7.9	10.0	13.1	15.4	15.4	11.8	11.8	*11.5*	< 0.004*
	10-19	M	26.7	28.6	30.1	30.8	28.6	30.4	27.0	31.6	*29.2*	< 0.014*

It could be speculated that the frequent use of drugs for ADHD in Nordland County represented an overuse of these drugs due to malpractice or deviation from national guidelines. We therefore performed a quality of care simulation comparing users born between January 1^st^ and June 30^th^ with those born between July 1^st^ and December 31^st^. However, we did not detect any significant differences indicating an overuse or deviation in northern Norway.

During study period the mean annual national cost of the ADHD drugs was €13,441,857 and the cost per user per year was € 919. The corresponding figures in the northern region were €1,587,883 and €885. Standardizing these figures according to population (northern region had 9.9% of the population, 2007) a total of €257,139 would have been saved annually if the national level of consumption had been achieved in northern Norway.

## 4. Discussion

In this study, we have documented that drugs for the treatment of ADHD are significantly more often prescribed to children and youths in northern Norway than in any other Norwegian health region. There were significantly more female users (age group of 10-19 years) in the northern region. Nordland County was the “culprit”. In this county, drugs for the treatment of ADHD were significantly more often prescribed than in the other counties of northern Norway. There were no regional differences between subgroups based on month of birth.

ADHD affects around 1-3% of children and have been reported affecting between 3-5% of school aged children (Biederman et al., 2004). In a recent published Norwegian study, the incidence was 2% among children aged 6-12 years (Surén et al., 2013). Figures in literature show that almost 0.5% of children aged 0-9 years and 2% of those aged 10-19 years were users of drugs for the treatment of ADHD (MTA, 1999). In a German study (Garbe et al., 2012), half of patients received ADHD drug treatment within a follow up time of up to four years.

Internationally, male/female ratio has been reported 4:1 (MTA, 1999). This is in coherence with our national and regional figures among the age group of 0-9 years. A Norwegian study concluded recently a rate of 2.8:1 among the age group 6-12 years (Surén et al., 2013). Whereas figures in adolescence internationally have been reported between 4:1 and 9:1 (MTA, 1999), we disclosed significantly lower figures (2.2:1) in our region. Our findings were even lower than the prior study by Åsheim and colleagues (2007). Based on data from Nordland County during the time period 1999-2004 they revealed boys constituting 78% of patients below the age of 18. In this context, the high share of girls in our survey is remarkable and should be further investigated.

Possible risk factors for ADHD are genetic factors, alcohol use and smoking habits during pregnancy and psychosocial factors among parents. Smoking during pregnancy has been documented increasing the risk of hyperkinetic disorder in offspring (Linnet et al., 2005). This Danish study concluded that women who smoked during pregnancy had a 3-fold increased risk for having offspring with hyperkinetic disorder compared with nonsmokers. The percentage of smokers during pregnancy is higher in northern Norway and it was most common in Finnmark (Norum et al., 2013). As Nordland County had the lowest percentage of smokers during pregnancy (in northern Norway), the findings by Linnet and colleagues (2005) cannot explain our findings. We therefore suggest that especially genetics and psychosocial factors should be topics in future studies. Furthermore girls with ADHD and their parents could be informed about a reported increased risk of alcohol abuse. Dalsgaard and colleagues (2014) concluded their results warrant increased focus on the possibly increased risk of substance abuse in females with ADHD compared to males with ADHD.

A high prescription rate in northern Norway and especially in Nordland County does not necessary mean a higher incidence of ADHD. However, the higher incidence of ADHD in northern Norway and especially in Nordland has been documented in a prior study (Surén et al., 2013). Surén and colleagues (2013) reported that Nordland County (3.3%) had the second highest incidence levels of ADHD (children aged 6-12 years) among all Norwegian counties. We cannot explain the high figures of Nordland County. ADHD is an underlying neurological problem where incidence rates should not have correlation or causal relation to the date of birth (Evans et al., 2010). Whereas Kowalyk and colleagues (2012) did not reveal any link between date of birth and ADHD symptoms in adults, other studies have indicated differences among children (Evans et al., 2010, Morrow et al., 2012; Elder, 2010). Age relative to peers in class and the resulting differences in behaviour may directly affect child’s probability of being diagnosed with and treated for ADHD (Evans et al., 2010, Morrow et al., 2012). Elder (2010) indicated that many diagnoses are driven by teachers’ perceptions of poor behaviour among the youngest children in a classroom. In British Colombia, girls born in December were 70% more likely to receive a diagnosis of ADHD than girls born in January (Morrow et al., 2012). The corresponding figure among boys was only 30%. Similarly, girls were 77% more likely and boys 41% more likely to be given a prescription for a medication to treat ADHD if they were born in December than if they were born in January. Despite a higher rate of females in our region, we could not detect any deviation between the northern region and the others. Furthermore, we had no data on gender connected to exact date of birth. Consequently we could not run further sub-analysis on gender.

Differences between Troms, Finnmark and Nordland County may partly be explained by organizational structure. Norwegian researchers have mentioned that a high number of units (29 hospitals and 102 child and adolescent psychiatry units) may cause limited experience in the diagnosis and care of ADHD patients in the small units (Surén et al., 2013; Groholt, 2013). In northern Norway, there are two major institutions in the psychiatric health care, the University hospital of North Norway (UNN) and the Nordland hospital (NH). Furthermore, there are in total 17 out-patient specialist units taking care of diagnosis and treatment. UNN support Finnmark with its psychiatric hospital and there is a strong cooperation between the UNN and the Finnmark hospital trust. This may explain their similar treatment culture. However, the UNN and NH have separate institutions and the cooperation between UNN and NH has been limited. Recently, a regional unit for patient safety in clinical care was initiated at NH. The development and implementation of quality management tools is an important part of this unit’s future activity and we believe this unit may give valuable support in future research. In the future, updated national treatment guidelines from the Norwegian Directorate of Health (www.helsedirektoratet.no), audits and the National Centre of Competence (www.nasjkomp.no) may also be important partners in ensuring an equal quality of care of ADHD-patients throughout the country.

The monthly willingness to pay (WTP) for an ADHD drug generating full effectiveness, no side-effects and once-daily dosing was estimated € 790 for adolescents and € 360 for adults in a Scandinavian study (Glenngård et al., 2012). We calculated a national cost of ADHD drugs per user of € 77/user per month. However, these costs were covered by the National health insurance. We had no individual data on the mean time of ADHD drug therapy. A Danish study (Pottegård et al., 2013) reported the mean treatment duration among children who had their medical therapy initiated before the age of 13 years varied between 3.6-4.2 years. In this context our figures are obviously below Scandinavian patients WTP limits.

During study period, the annual total ADHD drug cost was raised (undiscounted) by 65% from €8.3 million (2004) to €13.7 million. This was lower than the figures reported by Åsheim and colleagues (2007). They revealed that sale of stimulants in Nordland County defined as daily doses had increased 5-fold from 1999 to 2004. Furthermore, they concluded that this increase had to be viewed in the light of the increased number of physicians working in child psychiatry coupled with the development of better neuropsychological services. However, we would also mention that the last decade´s campaigns, by pharmaceutical companies to publicize the syndrome and promote the pills, should not be forgotten. These drugs should not be portrayed as “benign medications safer than aspirin”. They can have significant side effects and are regulated in the same class as morphine and oxycodone because of their potential for abuse and addiction. We therefore argue that prescription databases should be employed in monitoring the use of these drugs and alternative strategies of therapy should continuously be kept in mind.

## 5. Conclusions

Based on the NPD we have documented that drugs for the treatment of ADHD are more commonly prescribed in northern Norway. High figure in Nordland County and a significant share of females treated with these drugs in our region were revealed. In the future, the national prescription database should be employed in further quality of care analysis and in the monitoring of the treatment for ADHD in Norway. Furthermore, the high rate of females in northern Norway undergoing medical treatment should be further elucidated.
